# Validating the use of dermal secretion as a matrix for monitoring glucocorticoid concentrations in African amphibian species

**DOI:** 10.1093/conphys/coz022

**Published:** 2019-05-13

**Authors:** Juan Scheun, Dominique Greeff, Katarina Medger, Andre Ganswindt

**Affiliations:** 1 Research & Scientific Services Department, National Zoological Garden, South African National Biodiversity Institute, Pretoria, Republic of South Africa; 2 Mammal Research Institute, Department of Zoology and Entomology, University of Pretoria, Pretoria, Republic of South Africa

**Keywords:** ACTH challenge, amphibians, dermal, glucocorticoids, physiological, urinary

## Abstract

The complex interaction between factors leading to amphibian declines is responsible for the inability to develop robust, standardized conservation action plans. Monitoring physiological stress responses in amphibians may provide an ideal tool to assist conservationists in this regard. This study aimed to validate dermal secretions as a robust matrix for monitoring glucocorticoid alterations in an African amphibian, the edible bullfrog (*Pyxicephalus edulis*). Both a biological (handling) and physiological (ACTH challenge) validation were conducted to determine which of five available enzyme immunoassays (EIAs) tested is most suited for monitoring alterations in dermal glucocorticoid (dGC) concentrations. Additionally, the most optimal body region for monitoring dGC concentrations in *P. edulis* was identified. To confirm the activation of the hypothalamic–pituitary–interrenal (HPI) axis following ACTH administration, urinary glucocorticoid metabolites (uGCM) were also quantified. The tested corticosterone EIA was the only assay able to monitor alterations in dGC concentrations following the handling event in *P. edulis*. Further validation during the ACTH challenge supported the corticosterone EIA in this regard. Dermal secretions collected from both the ventral and dorsal regions were suitable for monitoring dGC concentrations in both sexes. A considerable increase in uGCM concentrations following ACTH administration was found, suggesting that the dGC concentrations observed were derived from the HPI axis. This study offers further support for the use of dermal secretions as an important matrix for monitoring physiological stress, and thus general health, in amphibian species.

## Teaser

Amphibians are experiencing drastic population declines. New semi-invasive conservation methods are required to protect threatened species. The collection of mucus, from dorsal and ventral body regions, is an ideal matrix for monitoring stress hormones, and thus physiological stress, in African amphibians. This technique will assist in amphibian conservation in Africa.

## Introduction

In 2004, a global amphibian assessment indicated that 32% of global amphibian species are threatened with extinction, with a further 22.5% of amphibian species listed as data deficient and likely to be added to this list (Stuart *et al.*, 2004). Although a number of factors have been reported as important drivers of such decline, Blaustein and Kiesecker (2002) argued that the decline in amphibian populations within a specific habitat is not the result of a single factor, but rather the different biotic and abiotic factors acting in a context-specific manner, which could lead to amphibian declines that vary temporally and spatially. As a result of such complexity, arguing for a single stressor or risk factor as the main cause of population declines may be too simplistic and lead to the inability to develop robust, standardized conservation action plans for amphibians on a global scale (Beebee and Griffiths, 2005). In order to assist law-makers, managers and conservationists in developing amphibian conservation protocols which are context-dependent, the development and validation of conservation tools that can be used in a variety of environments and species are urgently needed.

In amphibians, the hypothalamic–pituitary–interrenal (HPI) axis functions in the same way as the hypothalamic–pituitary–adrenal (HPA) axis in mammals, that is, the production of glucocorticoids (GCs) in response to a perceived stressor (Denver *et al.*, 2002). Thus, similar to mammals, GCs can be used as important markers to determine the level of physiological stress experienced by an amphibian (Narayan, 2013). In contrast to many mammal species, which secrete cortisol as a major GC during the stress response, research has found corticosterone to be the major GC secreted by the HPI axis in amphibians (Narayan *et al.*, 2010; Gabor *et al.*, 2013; Graham *et al.*, 2013; Narayan *et al.*, 2013; Barsotti *et al.*, 2017). Despite the benefits of GC secretion in an organism, which include the necessary adjustments in metabolism, energy availability, enhancing cardiovascular activity and altering behaviour, all in order to restore homeostasis (Moberg, 2000; Romero, 2002), chronic secretion of GCs can have a number of deleterious effects. These include the suppression of the immune and reproductive systems, muscle atrophy, growth suppression and a shortened lifespan (Möstl and Palme, 2002; Sapolsky, 2002; Charmandari *et al.*, 2005; Cohen *et al.*, 2007). As such, monitoring GC patterns within endangered amphibians could offer important insight into amphibian health and wellbeing.

Although blood offers a robust matrix for monitoring ‘real-time’ GC hormone patterns in an individual, the difficulties associated with animal capture and restraint, along with a possible GC feedback as a result of the procedure (Romero and Reed, 2005), renders this approach impractical in many captive and free-ranging environments (Heistermann, 2010). In contrast, non-invasive hormone monitoring techniques through the collection and analysis of urine, faeces, hair and saliva allow for the measurement of GC metabolite concentrations with limited to no need for animal handling and capture (Heistermann, 2010; Sheriff *et al.*, 2011). However, it is important that all non-invasive stress analyses are validated to ensure that GCs, and their metabolites, monitored within the chosen matrix, reflect biologically relevant signals (Palme *et al.*, 2005). Here, validation refers to the artificial activation of the HPI axis in amphibians, resulting in an increase in circulating GC concentrations (Moore and Jessop, 2003). Such an activation can be obtained via the injection of synthetic adrenocorticotropic hormone (ACTH Challenge test; Touma and Palme, 2005; Sheriff *et al*., 2011) or through exposure to a presumed stressor such as capture, handling or transport stress (Biological validation; Narayan *et al.*, 2012; Baker *et al.*, 2013; Hämäläinen *et al.*, 2014; Santymire *et al.*, 2018). The collected samples are then analysed with a number of enzyme immunoassays (EIAs) in order to determine the most applicable test system for monitoring GC metabolite concentrations in the chosen matrix. In amphibians, the use of an ACTH challenge (Narayan *et al*., 2010; Barsotti *et al*., 2017; Santymire *et al*. 2018) as well as biological stressors, such as disease (Gabor *et al*., 2013) and handling (Narayan *et al*., 2013; Santymire *et al*. 2018), have resulted in the activation of the stress response and a significant elevation in GC secretion. As such, both biological and physiological validation techniques can be employed in amphibian species.

A number of studies have successfully validated the use of urine and faeces as matrices for monitoring hormone patterns in amphibian species (Szymanski *et al.*, 2006; Graham *et al*., 2013; Hogan *et al.*, 2013; Narayan, 2013; Cikanek *et al.*, 2014). Although these non-invasive hormone-monitoring techniques have offered amphibian conservationists a robust and reliable tool for assessing reproductive and adrenocortical activity, it is not always practical within both the captive and free-ranging environments. The small body size of many amphibian species (e.g. Pickersgill’s reed frog, *Hyperolius pickersgilli*, 22–30 mm in length) makes the collection of sufficient urine and faeces improbable. Further, many amphibians have extended gut passage times (>33 h; Jiang and Claussen, 1993), as such, collecting faecal samples from free-ranging individuals may require extended field observation periods to obtain a single excreted sample, making repeated sampling difficult and time-consuming.

A new non-invasive hormone monitoring technique is required to aid researchers and conservationists in collecting easy, longitudinal hormone data within the captive and free-ranging environments. As an initial step, Santymire *et al*. (2018) recently described the use of dermal secretions as a viable matrix for monitoring GC levels in a number of amphibian species. In that study, peak glucocorticoid concentrations, following ACTH administration, were observed between 30–90 min in a number of amphibian species. Although the study showed that adrenal activity can be measured in amphibian dermal secretion through an appropriate EIA, a number of questions remain unanswered. As the majority of amphibians excrete mucus across their entire body (Parakkal and Matoltsy, 1964; Fox, 1983), the most ideal area for monitoring dermal glucocorticoid (dGC) concentrations has not been determined, nor the rate of mucus excretion for that specific area. Additionally, as the skin is able to produce and excrete GCs (Santymire et al. 2018), the source of monitored GCs (skin vs HPI axis) needs to be confirmed through the analysis of a matrix known to be viable for monitoring HPI axis activation (e.g. urine; Narayan and Hero, 2010; Graham, et al., 2013).

*Pyxicephalus edulis* is a bullfrog (family: Ranidae) found within several bushveld vegetation types throughout sub-Saharan Africa, especially in low-lying areas that become flooded following heavy, seasonal-dependent rainfall (Jacobsen, 1989; Vlok *et al.*, 2013). The species spends the majority of the year underground (fossorial) until environmental conditions improve. Reversed sexual dimorphism is present in the species, with females being considerably larger than their male counterparts (Braack and Maguire, 2005). Similar to the majority of amphibian species, *P. edulis* has a tougher dorsal region which limits evaporate water loss, with the majority of water loss, and absorption, occurring through the ventral body region (JØrgensen, 1997; Lillywhite, 2006). Despite being utilized as a food source and the degradation of their natural habitat, *P. edulis* is currently listed as least concerned by the IUCN Red List of Threatened Species (2016). The relative abundance of the species, along with the ecological similarities it shares with a large number of amphibians (Cook and Minter, 2004), make *P. edulis* an ideal study species.

In this study, we aim to (i) validate the most appropriate EIA for measuring dGC concentrations in *P. edulis*, (ii) determine the most suitable body region to monitor dGC concentrations and (iii) determine dermal secretion rates of the study species.

## Materials and methods

### Study site and animals

The study was conducted on 31 *P. edulis* individuals housed at the South African National Biodiversity Institute’s National Zoological Garden (NZG), Pretoria, South Africa (25.73913° N, 28.18918° E) as part of its animal collection. The animals were housed individually in transparent plastic containers (30 cm × 20 cm), containing a dark plastic cup as a hide. To keep individuals hydrated, the container was lined with permeable aquarium filter foam to which clean, non-chlorinated water (24°C) was added. This ensured that *P. edulis* individuals remained hydrated throughout the study period while limiting the area of contact with water and the possible loss of dermal secretion. The water within each container was replaced when dirty/murky or when water loss through evaporation resulted in no water reaching the individual through the foam. A dry area was also included in the container should individuals need a respite from the moist surface. All individuals were allowed 15 days to acclimatize to their new environment prior to the beginning of the biological and physiological validation period. Individuals were fed according to a fixed schedule; we ensured feeding and sample collection events did not occur on the same day. All individuals were housed in the Endocrine Laboratory, NZG, at a constant temperature of 24 ± 1°C [median ± standard deviation (SD)]. The entire study was performed with the approval of the NZG Animal Use and Care Committee (NZG/RES/P16/19).

### Dermal secretion rate

A pilot study was conducted to determine the dermal mucus secretion rate for both the dorsal and ventral regions under varying time intervals. Here, 24 individuals of unknown sex were taken from their containers, wiped clean with a wet cloth and placed into individual plastic enclosures containing no foam, hide or water. Individuals were divided into three groups (*n* = 8 for each) with different blotting intervals (10, 20 and 30 min; repeated six times). To determine whether moisture was present or absent at each defined interval, a small section (1 cm × 1 cm) of the ventral and dorsal region was blotted with a single-ply Kimwipe (Kimtech Science Brand, Kimberley-Clark Professional, USA). Dermal mucus was defined as present if a clear sign of mucus was seen (wet spot) on the Kimwipe following blotting. In contrast to this, dermal mucus was defined as absent if no sign of mucus secretion was observed on the Kimwipe after blotting. The period of optimum dermal secretion production was defined as any time interval where more than 75% of individuals within a group had dermal secretion present. An individual was removed from the treatment group when it urinated in its container and came in contact with the urine to avoid any confounding effects.

### Sample collection

#### Dermal secretion

The collection of dermal secretions was conducted according to a predetermined sampling protocol for both the biological and physiological validation performed during the study. All animals were handled with clean, decontaminated equipment and fresh, disposable gloves. Individuals were held in one hand and gently but firmly swabbed (2 mm-diameter plastic cotton swab without adhesive; Citoswab® transport swab, 2120-0015, Haimen City, China) three times on the chosen body (dorsal/ventral) surface along a length of ~2.5 cm. The body region swabbed first was randomized for each swabbing event (dorsal-ventral/ventral-dorsal). In an attempt to reduce cross-contamination and the removal of dermal secretion, care was taken not to come in contact with the dorsal or ventral body region during the sampling event. Sampled individuals were placed back into their respective containers. Individual swabs were placed into a 2 ml microcentrifuge tube containing 1 ml of 70% ethanol and sealed with Parafilm (Bemis Company, Inc., Oshkosh, USA) to reduce potential leakage and evaporation. All microcentrifuge tubes were placed at −20°C until further processing.

#### Urine

Urine samples were collected during the physiological validation period of the study. To minimize the need for prolonged animal handling, urine was collected in unison with dermal secretions when present. Here, individuals were held above a clean, decontaminated container during dermal secretion collection, allowing for excreted urine to be collected. All urine samples were transferred into individual microcentrifuge tubes, sealed with Parafilm and stored at −20°C until analysis.

### Experimental design

#### Biological validation

To determine whether GC concentrations could be detected in dermal secretion samples from *P. edulis,* a biological validation was conducted on six individuals of unknown sex. Here, animal handling and restraint were used as a biological stressor. The six individuals were divided into two groups where either dorsal (*n* = 3) or ventral (*n* = 3) swabs were collected. Individuals were removed from holding containers and dermal secretion samples were collected immediately (time 0; baseline sample). Individuals were then handled continuously for 3 min before being placed back into their individual containers. Following this, dermal secretion samples were collected at 5 min intervals until 20 min post-stressor, and a final sample was collected at 60 min post-stressor. A total of six samples were collected per individual.

#### Physiological validation

The physiological validation was conducted on 16 female (F) and 15 male (M) *P. edulis*. The 31 *P. edulis* individuals, none of which formed part of the biological validation group, were randomly assigned to one of two groups, namely a control (8M, 8F) or ACTH group (7M, 8F).

Dermal secretion and urine were collected from all 31 study animals at 07h00 daily for 3 consecutive days to determine baseline dGC and urinary glucocorticoid metabolite (uGCM) concentrations. At 06h00 on the fourth day, each of the ACTH animals (7M,8F) were injected intraperitoneally with 1 IU/kg long-acting, synthetic ACTH (0.443 μg/g of Synacthen®, 1-24 human, rat, Novartis, South Africa Pty Ltd) in 0.1 ml frog Ringer’s solution. This ACTH dose was chosen as it has been used successfully in a number of frog species to evoke a stress response (Narayan and Hero, 2010; Barsotti *et al*., 2017; Hammond *et al.*, 2018). The control animals (8M/8F) were injected with 0.1 ml Ringer’s solution intraperitoneally. As a result of previous findings in time to peak dGC concentrations following ACTH administration (Santymire *et al*., 2018), as well as the long-acting nature of the chosen ACTH, the first dorsal and ventral dermal secretion, as well as excreted urine samples, were collected 45 min after ACTH administration from all 31 individuals. Thereafter, dermal secretion and urine samples were collected at 3 h intervals over 2 days.

### Steroid extraction

The extraction process for dermal secretion samples was conducted according to Santymire *et al*. (2018). In brief, dermal secretion samples were kept at room temperature for 30 min prior to the start of the extraction process. The samples were then shaken on a water bath shaker at 70 rpm for 5 min before being briefly vortexed. Following a 15 s centrifuge spin down (1500 *g*), 0.5 ml of each sample was transferred into a new, pre-labelled 2 ml microcentrifuge tube. The extracts were then placed into an incubator oven at 60°C until dry (~5 h). Two to three glass beads were added to the dried extract of each tube prior to the addition of 0.5 ml assay buffer. Following the vortex of each sample for 15 s at maximum speed, samples were placed into a sonicator for 20 min. Finally, the samples were shaken on a water bath shaker for 30 min at 70 rpm. Samples were stored at −20°C until analysis. Native urine samples were used for the analysis of uGCM concentrations.

### Steroid analyses

#### Biological validation

Dermal secretion extracts collected during the biological validation event (*n =* 36) were measured undiluted for immunoreactive dGCs using five EIAs: (i) an 11-oxoeatiocholanolone I (detecting 11,17-dioxoandrostanes), (ii) an 11-oxoaetiocholanalone II (detecting dGCs with a 5β-3α-ol-11-one structure), (iii) a 5α-pregnane-3β,11β,21-triol-20-one (measuring 3β,11β-diol cortisol metabolites), (iv) a cortisol and (v) a corticosterone EIA. The inclusion of assays using antibodies designed to measure cortisol or corticosterone as well as widely used group-specific EIAs (Palme, 2019), should deem appropriate to identify a reliable test system for monitoring GC metabolites in dermal secretion in our target species. Details of the five EIAs, including cross-reactivities, are described by Palme and Möstl (1997) for 11-oxoaetiocholanolone I and cortisol, Möstl *et al.* (2002) for 11-oxoaetiocholanalone II and Touma *et al.* (2003) for 5α-pregnane-3β,11β,21-triol-20-one and corticosterone. Assay sensitivity was 0.02 ng/ml for 11-oxoaetiocholanolone I, 11-oxoaetiocholanolone II and cortisol, while the corticosterone and 5α-pregnane-3β,11β,21-triol-20-one EIAs had sensitivities of 0.04 ng/ml and 0.008 ng/ml, respectively. Serial dilutions of extracted samples gave displacement curves that were parallel to the respective standard curves in all assays (<5% deviation in the slope). Intra-assay coefficient of variation (CV), determined by repeated measurements of high- and low-value quality controls were 4.04% and 4.77% for 11-oxoaetiocholanolone I, 3.29% and 5.62% for 11-oxoaetiocholanalone II, 6.62% and 6.70% for 5α-pregnane-3β,11β,21-triol-20-one, 4.64% and 5.96% for cortisol and 4.25% and 5.0% for corticosterone.

All samples analysed with the 11-oxoaetiocholanolone I, 11-oxoaetiocholanalone II and cortisol EIAs during the biological validation process displayed dGC concentrations below their respective linear ranges and were thus unable to successfully monitor mucosal GC patterns within dermal secretions. Similarly, <50% of the dermal secretion samples analysed with the 11-oxoaetiocholanalone II and 5α-pregnane-3β,11β,21-triol-20-one EIAs had dGC concentrations within the linear range. The corticosterone EIA was the only assay capable of measuring dGC concentrations in all dermal secretion samples. As such, all subsequent dermal secretion samples were analysed using the corticosterone EIA.

#### Physiological validation

A total of 558 dermal secretion samples were analysed using the corticosterone EIA (18 per individual; nine dorsal, nine ventral). The intra-assay CV was 4.15% and 5.41%, while the inter-assay CV was 10.19% and 11.20%.

The low rate of urine excretion during the swabbing events resulted in a limited number of samples collected from each study animal (range: 3–9); additionally, a number of samples had urine amounts below the minimum quantity required for uGCM analysis. As such, only a subset (*n* = 44) of urine samples from ACTH (2M, 2F) and control (2M, 2F) animals could be analysed. Urine samples were measured for immunoreactive uGCMs using four EIAs, namely 11-oxoaetiocholanolone I, 11-oxoaetiocholanalone II, 5α-pregnane-3β,11β,21-triol-20-one and corticosterone. Intra-assay CV of high- and low-value quality controls were 3.83% and 4.09% for 11-oxoaetiocholanolone I, 4.24% and 5.31% for 11-oxoaetiocholanalone II, 6.62% and 6.70% for 5α-pregnane-3β-11β-21-triol-20-one and 4.15% and 5.41% for corticosterone. Inter-assay CV of high- and low-value quality controls were 7.73% and 13.60% for 11-oxoaetiocholanolone I, 10.45% and 15.07% for 11-oxoaetiocholanalone II, 11.57% and 11.59% for 5α-pregnane-3β-11β-21-triol-20-one and 6.76% and 8.84% for corticosterone. Serial dilutions of extracted samples gave displacement curves that were parallel to the respective standard curves in all assays (<5% deviation in the slope). All enzyme immunoassay analyses throughout the study were performed on microtiter plates at the Endocrine Research Laboratory, University of Pretoria, as described by Ganswindt *et al.* (2012).

#### Urine-specific gravity calculations

To standardize uGCM results, specific gravity (SG) was performed on all 44 urine samples at Clinpath Laboratories, University of Pretoria, Pretoria, South Africa This was done by determining the ratio of urine specimen to water density in each urine sample (Carrieri *et al.*, 2000). Here, the formula used to standardize each hormone metabolite result was:

*SG-corrected concentration = raw hormone metabolite concentration x* $\frac{SGtarget-1.0}{\ \mathrm{SGsample}-1.0}$ (Miller *et al.*, 2004)

where SG_target_ is the population mean, in this case 1.002. The results were referred to as specific gravity corrected values and expressed as ng/mL.

### Data analysis

Differences in baseline GC concentrations, as well as the degree of GC secretion following a stressful event, exist between individuals (Dingemanse *et al.*, 2010; Koolhaas *et al.*, 2010). A circumstance, largely due to varying GC receptor densities within the brain, the interaction between GCs and the receptors, as well as previous life history experiences of an individual (Romero, 2004; Champagne *et al.*, 2008; Joëls and Baram, 2009). To account for such differences in reflected absolute dGC values and allow for the comparison between individuals, the percentage dGC response was calculated for each individual.

Peak percentage dGC response = (Peak dGC value/Baseline dGC value)*100.

Individual baseline dGC values were determined by calculating the median dGC values prior ACTH/saline injection.

Thus, a 100% (1-fold) increase was indicative of baseline value and no change in HPI activity. For statistical analyses, only individual ‘peak percentage dGC response’ was used for comparison between experimental groups and sexes. To determine whether a significant difference in peak percentage dGC responses exist between experimental groups and body regions, one-way analysis of variances (ANOVAs) were performed separately for each sex. A *post-hoc* Tukey test was performed where a significant difference was found during an ANOVA test. Similarly, a one-way ANOVA was conducted to determine whether a significant difference in dorsal and ventral ‘peak percentage dGC response’ was present between (i) male and female ACTH animals and (ii) male and female control animals.

To define whether a correlation between dGC concentrations excreted by the dorsal and ventral region exists, a generalized linear mixed model (GLMM) approach proposed by Hamlett *et al.* (2004) was performed. This test calculates the correlation estimate from within a subject variance matrix (individual ID as random factor; R package: nlme, MuMin).

One-way ANOVAs were conducted to determine whether a significant difference in time until peak dGC concentration (post-injection) occurred between body regions and experimental groups (ACTH, control) for each sex. To determine whether a significant difference in mucus production rates existed between the three established blotting groups, a GLMM was conducted, followed by an ANOVA (random factors: individual, sex; R package: lme4) for both the dorsal and ventral regions.

The α-level of significance was set at 0.05. As a result of the low number of samples, uGCM data are discussed through descriptive means. Analytical statistics were performed using R software (R 3.2.1, R Development Core Team 2013). Values are given as mean ± SD unless stated otherwise.

## Results

### Physiological validation: urine

Individuals were placed into their respective cages between swabbing events, which may have led to unobserved urination events. Thus, the exact time to elevate, peak and return to baseline uGCM response values could not be defined during this study for either male or female ACTH injected animals.

**Figure 1:**
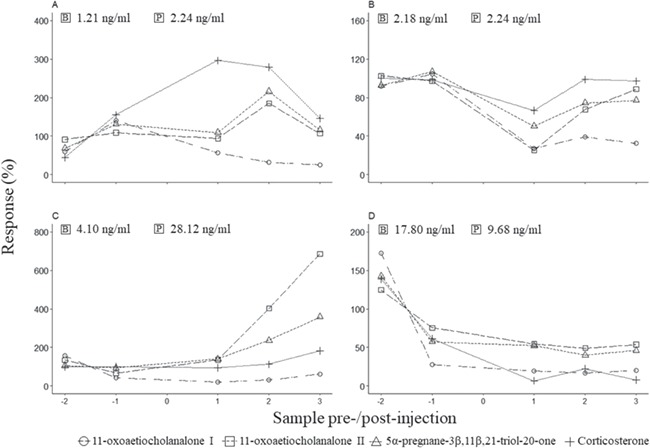
The percentage uGCM response in one ACTH injected female (**A**) and male (**C**), as well as one control female (**B**) and male (**D**). The ACTH and Ringer’s solution administration occurred at sample 0. Also shown is the absolute baseline (B) and peak post injection (P) uGCM concentrations for the most appropriate enzyme immunoassay chosen (11-oxoaetiocholanalone II).

For both ACTH injected females, three of the four EIAs showed an uGCM response exceeding 100% (Table 1). Here, the corticosterone EIA showed the highest response, followed by the 5α-pregnane-3β,11β,21-triol-20-one and 11-oxoaetiocholanalone II EIAs. For ACTH female 1, the peak uGCM response was found on the third and final collected sample (~25 h) post-injection; as such, we did not observe the uGCM levels return to baseline during the study. Peak uGCM concentrations for ACTH female 2 was observed on the first collected sample (~4 h) post-injection (Fig. 1A). Similar to ACTH female 1, uGCM response levels did not return to baseline during the observation period. Except for the 11-oxoaetiocholanolone I EIA in control female 1 (Table 1), no other EIA showed an uGCM response exceeding 100% for both control females (Fig. 1B).

**Table 1:** The peak urinary glucocorticoid metabolite response (percentage) in male and female individuals within the ACTH and control groups, across the four EIAs tested

ACTH group				
	11-oxoaetiocholanolone I	11-oxoaetiocholanalone II	5α-pregnane-3β,11β,21-triol-20-one	Corticosterone
Male 1	138.07	231.91	189.51	137.84
Male 2	60.36	685.95	358.57	181.93
Female 1	105.38	170.39	124.38	160.41
Female 2	56.97	185.13	216.07	296.23
Mean ± SD	90.20 ± 38.80	318.35 ± 246.47	222.13 ± 98.78	194.10 ± 70.42
Control group				
	11-oxoaetiocholanolone I	11-oxoaetiocholanalone II	5α-pregnane-3β,11β,21-triol-20-one	Corticosterone
Male 1	35.70	43.95	68.35	73.80
Male 2	20.48	54.40	52.16	22.19
Female 1	111.21	65.48	58.48	34.92
Female 2	3.93	88.66	77.16	98.83
Mean ± SD	51.58 ± 40.56	63.12 ± 19.16	64.04 ± 11.00	57.44 ± 35.26

The average response for each EIA is given as mean ± SD.

For both ACTH injected males, the uGCM response exceeded 100% in three of the four EIAs (Table 1). In this regard, the 11-oxoaetiocholanalone II showed the highest response, followed by 5α-pregnane-3β,11β,21-triol-20-one. For ACTH male 1, a peak uGCM response was observed at the first collected urine sample (45 min) following the ACTH administration, with uGCM response levels returning to baseline at the third collected sample (~7 h) post-injection. In contrast to this, ACTH male 2 showed a peak uGCM response at the third collected sample (~10 h) post-injection (Fig. 1C). As the peak response occurred during the final sample analysed, uGCM response levels did not return to baseline during our observations of this individual. No EIA showed a response exceeding 100% following only Ringer’s solution administration (Table 1, Fig. 1D).

#### Biological validation

The time of peak dGC concentrations following handling differed between regions, with peak samples observed after 15 ± 25 min and 20 ± 8 min for the dorsal and ventral regions, respectively. Dorsal samples collected from three individuals showed a 106 ± 4% increase in dGC concentrations, while ventral samples showed a considerably higher dGC concentration increase of 140 ± 19% from baseline values.

#### Physiological validation: dermal secretions

**Figure 2:**
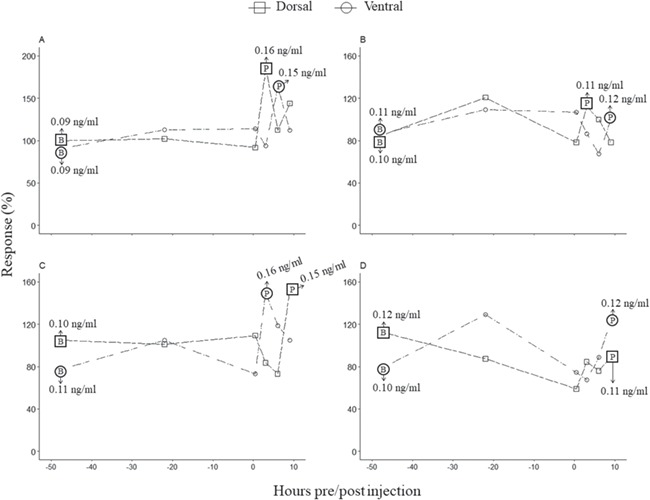
The percent dGC response for one ACTH injected female (**A**) and male (**C**), as well as one control female (**B**) and male (**D**), from both the dorsal and ventral regions. The ACTH and Ringer’s solution administration occurred at time 0. The calculated baseline (B) and peak post injection (P) absolute dGC concentrations are shown.

**Figure 3:**
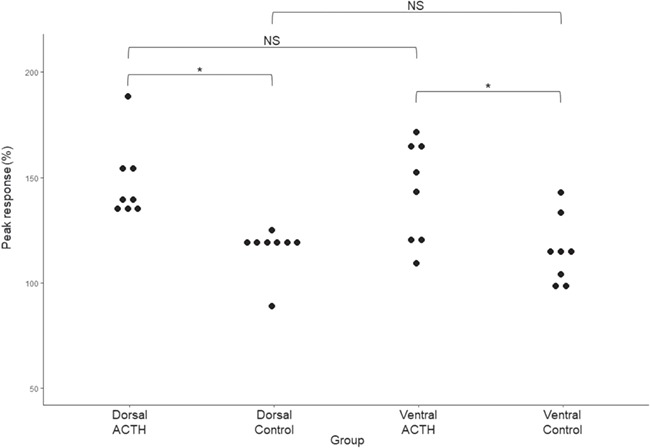
The peak ventral and dorsal dermal glucocorticoid response (percentage) following the ACTH and Ringer’s solution administration for all female individuals within the ACTH and control groups. Significance level between groups is indicated by * (*P* < 0.05), while non-significance (*P* > 0.05) is indicated by non-significance (NS).

A considerable increase in dGC concentrations was observed following ACTH administration in both male and female individuals compared to control animals (Fig. 2, Table 2). A significant difference in the peak percentage dGC response was observed between female ACTH and control groups (F_(3,26)_ = 6.62, *P* = 0.002, Fig. 3). A *post-hoc* Tukey HSD test showed that the mean percentage dGC response in ACTH dorsal (147 ± 18%) and ventral (143 ± 26%) regions were significantly higher (*P* < 0.05, Fig. 3) than their control counterparts (dorsal: 115 ± 11%, ventral: 115 ± 17%); however, no significant difference was found within experimental groups (dorsal vs ventral) (Fig. 3).

**Table 2:** The time of peak dGC sample collection, the percentage dGC response to the injection event and the normality of data from the respective ACTH and control groups

Male				
Group	Region	Number of peak dGC response samples	Time to peak dGC sample post-injection (hours)	Median peak percentage dGC response (post-injection)
ACTH	Dorsal	7	5.50 ± 3.37	139.13 ± 18.47
ACTH	Ventral	7	5.50 ± 2.13	133.33 ± 19.29
Control	Dorsal	8	7.00 ± 3.11	100.00 ± 16.90
Control	Ventral	8	4.00 ± 3.82	103.70 ± 37.14
Female				
Group	Region	Number of peak dGC response samples	Time to peak dGC sample post-injection (hours)	Median percentage dGC response post-injection (ng/ml)
ACTH	Dorsal	8	5.50 ± 3.37	139.47 ± 18.24
ACTH	Ventral	8	4.00 ± 2.94	152.38 ± 25.93
Control	Dorsal	8	7.00 ± 3.45	118.35 ± 11.09
Control	Ventral	8	4.00 ± 2.49	112 ± 17.34

Time to peak dGC concentrations ranged from 45 min to 10 h for both male and female animals. Values are given as median ± SD.

**Figure 4:**
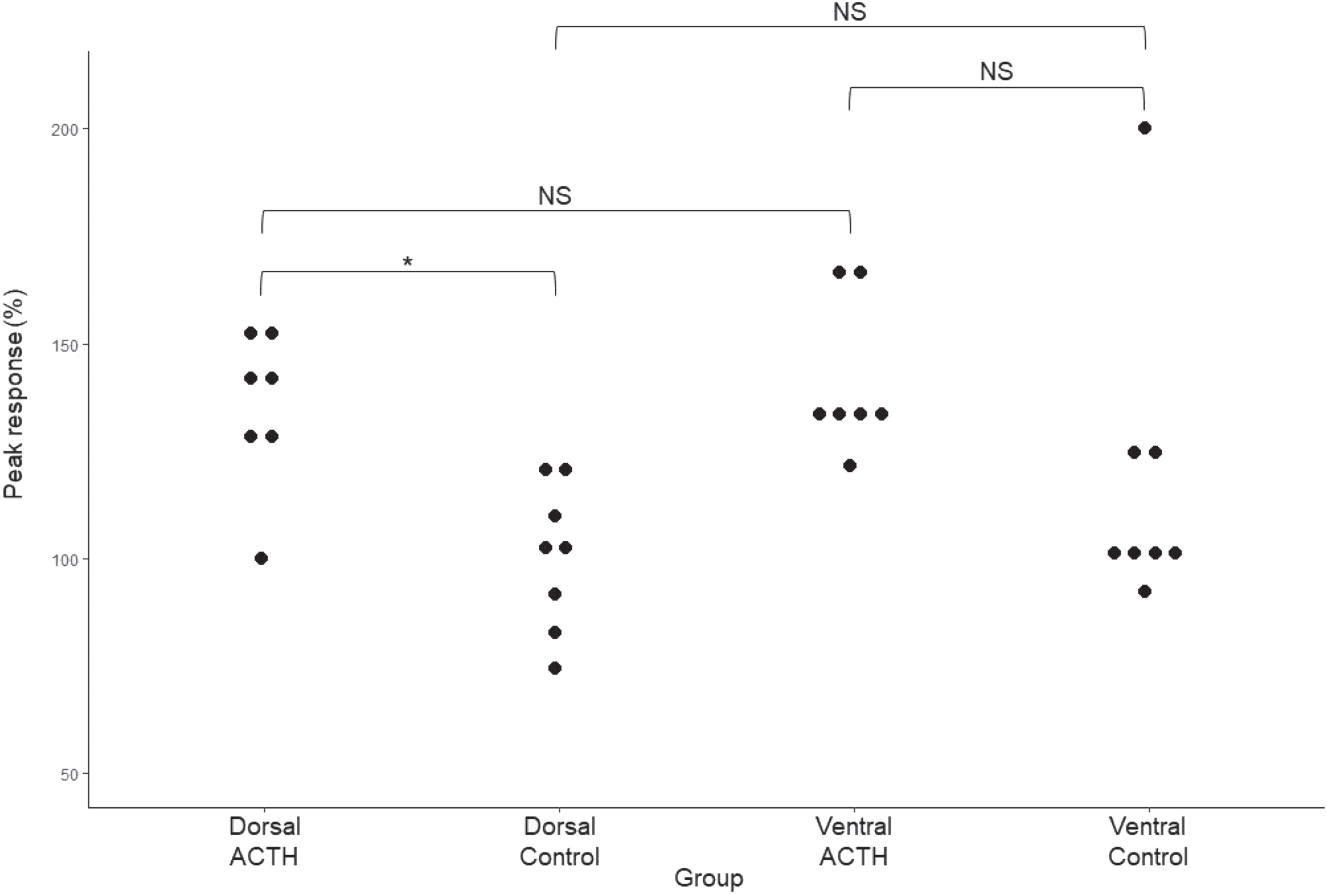
The peak ventral and dorsal dermal glucocorticoid response (percentage) following the ACTH and Ringer’s solution administration for all male individuals within the ACTH and control groups. Significance level between groups is indicated by * (*P* < 0.05), while non-significance (*P* > 0.05) is indicated by NS.

Similar to study females, a significant difference in the peak percentage dGC response was observed between male ACTH and control groups (F_(3,24)_ = 4.14, *P* = 0.017). A *post-hoc* Tukey HSD test showed that the mean percentage dGC response in the ACTH dorsal (135 ± 18%) region was significantly higher (*P* < 0.05, Fig. 4) than the control dorsal region (dorsal: 100 ± 17%). Despite no significant difference between ACTH ventral (142 ± 19%) and control ventral (121 ± 37%) regions, median ‘peak response dGC levels’ from ACTH ventral regions were considerably higher (Fig. 4) than their control counterparts. Lastly, no significant difference was found within male experimental groups (dorsal vs ventral, Fig. 4).

A one-way ANOVA showed no significant difference in the dorsal and ventral ‘peak percentage dGC response’ between ACTH males and females (F_(3,26_ = 0.52, *P* = 0.67) or between control males and females (F_(3,28)_ = 1.062, *P* = 0.38).

There was no significant correlation in the pattern of dGC secretion between body regions of all study individuals (*P* = 0.29, t = 1.06, df = 134, r^2^ = 0.04). Similarly, there was no significant difference in the time until peak dGC concentration (post-injection) in ACTH and control males (F_(3,23)_ = 0.711, *P* = 0.555) and females (F_(3,26)_ = 2.98, *P* = 0.69), across both body regions. Despite this, there was a considerable time range (45 min–10 h) within experimental groups as to when peak dGC samples were collected (Table 2).

#### Dermal secretion rate

Time interval did not play a significant role in determining dermal secretion rate on the dorsal region (F_(2,142)_ = 0.68, *P* = 0.50). However, time between swabbings, specifically at 30 min time intervals, was a significant factor for mucus production on the ventral body region (F_2,142_ = 3.83, *P* < 0.001), which showed an 85% dermal secretion presence in the 30 min swabbing interval group. The optimal dermal secretion mark of 75% set in this study was not seen at the 10 min (dorsal: 48%, ventral: 62%), 20 min (dorsal: 62%, ventral: 67%) or 30 min intervals (dorsal: 65%). Contamination by urine only occurred on the ventral body region throughout the experiment (10 min: 38%, 20 min: 27%, 30 min: 10%); these individuals were removed from further analyses.

## Discussion

This is the first study to validate the use of dermal secretions as a matrix for monitoring stress hormones in an African amphibian. Additionally, the study also determined the possibility of using various body regions for monitoring dGC concentrations in *P. edulis*. Finally, the study successfully described the time required for dermal secretion recovery following a swabbing event.

This study has successfully validated the most appropriate EIA for monitoring alterations in uGCM concentrations in *P. edulis*. Following the administration of ACTH to male and female animals, a considerable increase in uGCM concentrations was observed in three of the four EIAs used (124.38%–685.95%). In this regard, the 11-oxoaetiocholanalone II EIA showed the highest average uGCM response and seems to be the most suitable EIA for monitoring alterations in uGCM concentration in *P. edulis.* As a result of the urine sampling protocol employed during this study, the time to peak uGCM concentrations, and return to baseline levels, could not be determined. However, an initial increase in uGCM concentrations exceeding 100% was observed between 45 min and 7 h post-injection. In *P. edulis,* uGCM concentrations remained elevated at the end of the monitoring period in three of the four ACTH injected animals*.* A number of studies have shown individual- and species-specific variation in uGCM response following a stressor. For example, Narayan *et al*. (2010) showed that an initial increase in uGCM concentrations occurred 6 h after ACTH administration in the Fijian ground frog (*Platymantis vitiana*), with peak uGCM levels found between 1–4 days post-injection. In contrast to this, Narayan *et al*. (2012) demonstrated that peak uGCM concentrations occurred ~12 h post-stressor in the cane toad (*Rhinella marina*), with levels returning to baseline levels after ~24 h. Finally, the increase in uGCM concentrations following the ACTH injection indicates the activation of the HPI axis in amphibians. This supports the notion that the increase in dGC concentrations observed in this study following ACTH administration is a true reflection of the physiological stress experienced by *P. edulis* individuals.

The biological validation performed during this study assisted in determining the most suitable EIA for monitoring GC concentration in dermal secretions of *P. edulis*. Of the five EIAs tested, only the corticosterone EIA was able to do so in all dorsal and ventral samples. Although the increase in dGC concentrations following the handling event occurred almost immediately for all animals (15–20 min post-stressor), a considerable difference in time to peak dGC response following the handling event occurred between the six individuals. Goymann (2012) and Koolhaas *et al*. (2010) showed that the degree of HPA activation in response to a biological stressor (e.g. handling, injury or capture) may be individual-, species- and/or sex-specific. For amphibians, the variance in HPI activation was demonstrated by Narayan *et al*. (2012), where specific handling and sample collection protocols resulted in considerable variability in the stress response between individual cane toads (*R. marina*). This variability was also observed between male and female green frogs (*Rana esculenta*), with males having a substantially lower GC response than their female counterparts during the breeding period (Zerani *et al.*, 1991). The time to peak dGC concentrations following the handling event were similar to that found in eight species studied by Santymire *et al*. (2018), which included the green tree frog (*Hyla cinerea*), American toad (*Anaxyrus americanus*) and the northern leopard frog (*Lithobates pipiens*). However, as the time to peak dGC following the ACTH administration in this study was considerably longer (45 min–10 h), the origin of the monitored dGCs following the handling event needs to be considered. The skin contains both hormone receptors and the ability to produce hormones, making it an important endocrine organ (Zouboulis, 2009). The repeated stimulation of the skin, through swabbing, may lead to increased production of mucus and GCs from the skin, which may explain the rapid increase in dGC concentrations observed in this study. However, further research is required to confirm this.

The validity of the corticosterone EIA as the primary assay for monitoring dGC concentrations in *P. edulis* was further established through a physiological validation. The results from the ACTH challenge on all captive *P. edulis* confirm the ability of the corticosterone EIA to reliably monitor changes in GC concentrations as a measure of HPI activity using amphibian dermal secretions. There was no significant difference in dGC concentrations following ACTH administration between the dorsal and ventral region within sexes or experimental groups of this study. In the absence of such a difference, it would seem that the GC path from the HPI axis to both body regions is similar. To our knowledge, no information exists to support or deny such a claim. In spite of this, our results indicate that both regions can be used to monitor dGC concentrations as an indicator of physiological stress in the species.

Although the dGC concentrations in response to the ACTH administration was similar between body regions, we did not observe a significant correlation between regions, with time to peak dGC response a likely factor to account for this. The tougher dorsal integument may explain the difference in dGC excretion rate compared to the more permeable ventral region in the study animals (Lillywhite and Licht, 1974; Lillywhite and Maderson, 1988). However, the time to such peak dGC concentrations were extremely varied, which may explain why there was no significant difference in the time until peak dGC response between body regions. Although sex-specific differences in GC production, secretion and metabolism can be found between male and female amphibians (Narayan, 2013), there was no significant sex-related difference in dGC response to ACTH administration when using the chosen corticosterone EIA, reducing the notion of a likely sex-related difference in GC production and dermal secretion route in *P. edulis*.

The poikilothermic nature of amphibians may contribute to the delayed stress response, and variation to peak stress hormone concentrations, observed in this and other amphibian species (see Narayan *et al*. 2013). In contrast to the stress response being rapid in mammals and birds (>3 min; Romero and Reed, 2005), the slow metabolism inherent in amphibians (Bennett, 1978) may result in a longer than expected window to peak stress hormone secretion. It is this physiological trait which might explain the variation observed in a number of amphibians; i.e. small individual differences in the amphibian stress response, as a result of reproductive state, sex, previous experiences, etc. (Goyman, 2012), may result in longer delays to peak stress hormone concentrations between animals. Furthermore, it would appear that the activation of the stress response via ACTH administration in amphibians is considerably delayed (6 h–2 days) compared to biological stressors (e.g. handling; see Narayan, 2013), most likely as a result of the mentioned lower metabolic rates of poikilotherms. This extended time to peak-stress hormone levels, as well as the individual variation inherent in amphibians, should be considered when conducting non-invasive hormone monitoring techniques. Furthermore, repeated sampling following a stress event should be conducted to ensure the expected increase in uGCM/dGC concentrations is observed. Despite these possible pitfalls, the analysis of dermal secretion and urine as indicators of physiological stress in amphibians still represents an important tool to assist conservation efforts. Dermal secretion rates were considerably higher on the ventral compared to dorsal region of *P. edulis* across all blotting periods. Similarly, only the ventral region showed sufficient dermal secretion production (>75%) at 30 min blotting intervals. A number of factors are responsible for the dermal secretion rate in amphibians, such as the permeability of the skin, the prevailing environmental conditions, the adaptation of a species to its environment and the presence of specialized cells within the ventral region that allows for the rapid absorption and secretion of moisture (Toledo and Jared, 1993; Young *et al.*, 2005; Ogushi *et al.*, 2010). The latter may explain the high levels of mucus production observed in the ventral compared to dorsal region of *P. edulis*. Furthermore, *P. edulis* lives underground for most of the year and only comes above ground to breed. During that time, rainy periods may be followed by high temperatures in summer. Furthermore, the species is adapted to tropical and subtropical environments, where environmental temperatures are often high, while rainfall is limited to specific periods annually. Under such conditions, *P. edulis* may attempt to limit water loss. Although little is known on the adaptations bullfrogs have to limit water loss, similar traits found in other tropical and subtropical species may apply, which include (i) behavioural (body stance, shade seeking; Pough *et al.*, 1983; Connolly *et al.*, 2011), (ii) structural (a dorsal skin structure that limits water evaporation; Lillywhite and Maderson, 1988) and (iii) physiological (limited production and secretion of dermal mucus; Lillywhite, 1971; Toledo and Jared, 1993) parameters. Limited mucus production on the dorsal region may explain the pattern observed in *P. edulis* after repeated blotting and dermal stimulation. Despite the simplicity used in this study to determine the dermal secretion rate, and the limited information on *P. edulis* dermal structure and physiology, the information collected is important for a number of reasons. Firstly, should successive skin swabbings be required over a relatively short period, the information will ensure the formulation of an optimal sampling protocol. Secondly, the repeated production and excretion of dermal secretions specifically through the ventral body region can result in accelerated water loss in amphibians and should be taken into consideration when conducting such research (Lillywhite and Licht, 1974). Furthermore, the function of dermal secretions in the day-to-day survival of an individual includes respiration and water regulation, as well as anti-predator, anti-microbial and anti-fungal defence (Clarke, 1997). For example, the antimicrobial peptides found within dermal secretions play an important role in protecting amphibians against a number of pathogens, including the chytridiomycosis disease caused by *Batrachochytrium dendrobatidis* (Rollins-Smith, 2009; Rollins-Smith *et al.*, 2011). The removal of dermal secretion from a wild individual and its subsequent release into the natural environment prior to adequate dermal secretion replacement may lead to an increase in amphibian disease and mortality. Thus, species-specific dermal secretion rates should be determined prior to the use of this technique in the wild to ensure individual survival. Where possible, individuals should be allowed to replace lost moisture and dermal secretion layers in an enclosure with water prior to their release into the natural environment. Finally, although the ventral region showed a high rate of mucus secretion, the number of contamination events observed highlight the fact that care should be taken in using the region for sample collection in both captive and free-ranging conditions, especially as amphibian locomotion results in frequent contact between the substrate and ventral body region (Barclay, 1946). As such, the dorsal body region, despite its lower mucus production ability, may be the more robust region for mucus collection.

## Conclusion

A number of important findings resulted from the current study. Firstly, the study has successfully demonstrated the suitability of dermal secretions as a viable matrix for monitoring physiological stress in an African amphibian, supporting previous research by Santymire *et al*. (2018). Next, the excretion pattern of dGC concentration at different body regions in amphibians was determined, with the dorsal region being preferred as a result of limited contamination, as is found on the ventral body region. Finally, the most appropriate EIA for monitoring GC metabolite concentrations in urine and dermal secretions was established for *P. edulis*. This research has opened a number of new avenues that need to be followed to further strengthen this newly validated GC monitoring technique. Further research is required on (i) the mechanism involved in mucus production of both regions, (ii) the GC pathway in the dorsal and ventral body region (HPI axis–blood stream–dermal excretion) and (iii) the applicability of using such technique for long term GC monitoring in endangered amphibian in captive and free-ranging environments.
